# The Phase Distribution Characteristics and Interphase Mass Transfer Behaviors of the CO_2_–Water/Saline System under Gathering and Transportation Conditions: Insights on Molecular Dynamics

**DOI:** 10.3390/molecules29174256

**Published:** 2024-09-08

**Authors:** Shuang Wang, Qinglin Cheng, Zhidong Li, Shaosong Zhao, Yue Liu

**Affiliations:** 1 Key Lab of Ministry of Education for Enhancing the Oil and Gas Recovery Ratio, Northeast Petroleum University, Daqing 163318, China; 2 Daqing Oilfield Design Institute Co., Ltd., Daqing 163712, China

**Keywords:** oil and gas gathering and transportation, CO_2_–water system, molecular dynamics, interphase mass transfer, phase distribution characteristics

## Abstract

In order to investigate the interphase mass transfer and component distribution characteristics of the CO_2_–water system under micro-scale and nano-scale transport conditions, a micro-scale kinetic model representing interphase mass transfer in the CO_2_–water/saline system is developed in this paper. The molecular dynamics method is employed to delineate the diffusion and mass transfer processes of the system’s components, revealing the extent of the effects of variations in temperature, pressure, and salt ion concentration on interphase mass transfer and component distribution characteristics. The interphase mass transfer process in the CO_2_–water system under transport conditions can be categorized into three stages: approach, adsorption, and entrance. As the system temperature rises and pressure decreases, the peak density of CO_2_ molecules at the gas–liquid interface markedly drops, with their aggregation reducing and their diffusion capability enhancing. The specific hydration structures between salt ions and water molecules hinder the entry of CO_2_ into the aqueous phase. Additionally, as the salt concentration in water increases, the density peak of CO_2_ molecules at the gas–liquid interface slightly increases, while the density value in the water phase region significantly decreases.

## 1. Introduction

In oil and gas production, carbon dioxide flooding is widely employed to enhance oil and gas recovery [1,2]. However, in the later stages of development, this oil displacement method has also revealed significant issues, such as gas channeling and high flow rates, which impact the production efficiency and economic benefits of oil and gas fields. To address these challenges, researchers have developed gas-alternating-water flooding technology, designed to mitigate gas channeling and high flow rates through the synergistic interaction of water and carbon dioxide, thereby further enhancing oil recovery [3,4]. However, as the extracted liquid is raised to the surface for gathering and transportation, fluctuations in the external environment such as temperature and pressure can influence the interaction between the large volume of injected carbon dioxide and water, consequently modifying the mass transfer processes between the gas and liquid phases, which impacts the normal gathering and transportation of the extracted liquid [5,6]. Therefore, it is essential to study the interaction and mass transfer processes between carbon dioxide and water under surface gathering and transportation conditions.

At present, the research on the CO_2_–water system mainly focuses on the properties of two-phase equilibrium and mixed systems, and is generally studied through experimental and thermodynamic theories [7,8,9,10]. However, there is no thermodynamic model that can accurately predict the entire pressure and temperature range [7,11,12,13]. At the same time, it can be seen that macroscopic experimental studies have difficulty testing complex temperature and pressure conditions and fail to reveal the essence of the interaction between carbon dioxide and water molecules.

Molecular simulation is a principal research method for unveiling the essence of thermal phenomena between substances, penetrating into microscopic interaction mechanisms from a molecular level, thereby accounting for macroscopic phenomena and identifying pertinent properties [14,15,16,17]. Zhang et al. [18] use molecular dynamics to simulate the evolution of the water–CO_2_ interface at 300 K, as pressure shifts from low to critical levels. They observe that the growth of CO_2_ film at the interface is divided into two stages, with minimal alteration in the water surface structure, and the CO_2_ film has no effect on the orientation of water molecules at the interface. Shiga et al. [19] utilize molecular dynamics to explore the mechanisms by which temperature impacts the interfacial tension of CO_2_–water across an extensive temperature and pressure range (278–573 K, 8–50 MPa). At elevated temperatures, the interfacial tension between CO_2_ and water significantly reduces, with this temperature dependency directly linked to the excess molar entropy at the interface. Tulegenov [20] provides a more profound exploration of the interactions between water and carbon dioxide. The interaction energies of water–CO_2_ complexes are computed using methods such as MP2, CCSD, and CCSD(T). The findings reveal that electrostatic and dispersion energies are principal in the binding of molecules. Thus, molecular dynamics simulation offers a more profound description of the microscopic behaviors of carbon dioxide and water molecules, elucidating their microstructures and dynamical processes.

In summary, the current microscopic research on CO_2_–water system mainly focuses on the study of phase interfaces and interphase interactions, while there is relatively little research on the diffusion and mass transfer processes of each component in the CO_2_–water two-phase system. Moreover, the existing research conditions are mainly high-temperature and high-pressure conditions in underground reservoirs, and there is still precious little research on the diffusion and mass transfer processes of each component in the CO_2_–water two-phase system under surface gathering and transportation conditions [21,22]. The mass transfer process between phases and the structural characteristics of phase components in the CO_2_–water system are of great significance for the stable collection and transportation of produced liquid. Therefore, molecular dynamics is adopted to study the microscopic mass transfer process of CO_2_–water under gathering and transportation conditions in this paper, as well as exploring the structural and dynamic characteristics of each component in multiphase systems and revealing the influence laws of temperature, pressure, and salt components on the mass transfer and structural characteristics of each component.

## 2. Results and Discussion

### 2.1. Distribution Characteristics of Each Phase in C-W System

**Figure 1:**
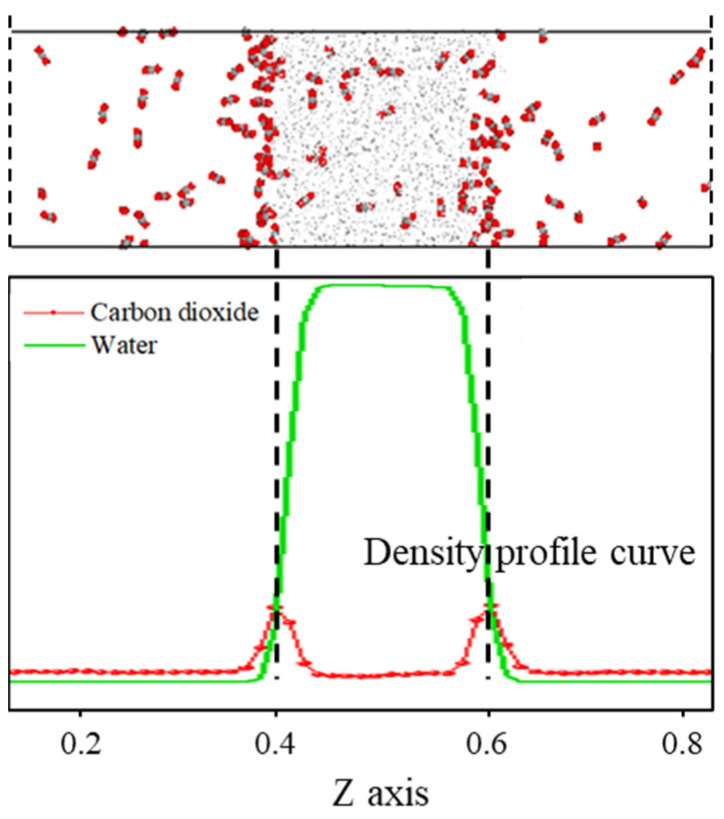
The distribution characteristics of the C-W system after equilibrium.

As shown in Figure 1, the density distribution curve of CO_2_ molecules exhibits two distinct peaks at the *z*-axis coordinates of approximately 0.4 and 0.6, with a relatively gentle trough near the center of the *z*-axis. In contrast, the density distribution curve of water molecules displays a prominent peak near the center of the *z*-axis, which is significantly larger than the density peak of CO_2_. This density distribution pattern is consistent with the molecular distribution observed in the final frame after system equilibration. The peaks in the CO_2_ density distribution curve arise from the sudden increase in density caused by the formation of a certain thickness of adsorption layer at the interface, while the valleys are due to a significant decrease in the number of CO_2_ molecules in the aqueous phase compared to the adsorption layer. The prominent peak in the water molecule density distribution curve at the center of the *z*-axis is attributed to the strong liquid properties of water, characterized by larger intermolecular forces and tighter ordering, which concentrates the density curve and distributes its peak at the center of the *z*-axis.

### 2.2. Effects of Temperature and Pressure on Phase Composition Structure and Diffusion Characteristics of C-W Two-Phase System

**Figure 2:**
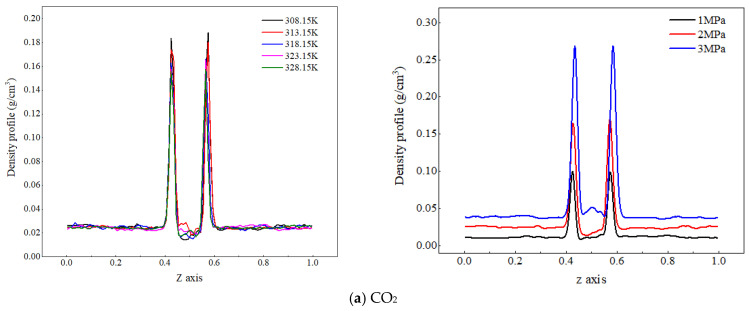

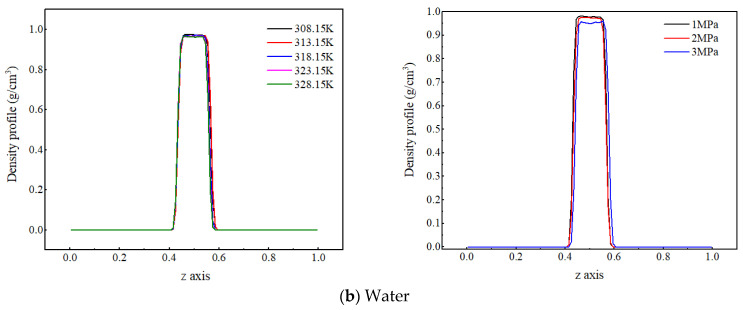
Density distribution of CO_2_ and water molecules at different temperatures and pressures.

Due to the significant difference in density between gaseous CO_2_ and liquid water, in order to facilitate the analysis of the effects of temperature and pressure on the spatial distribution characteristics of each component in the CO_2_–pure water two-phase system, the density distribution of CO_2_ molecules and water molecules under different temperatures and pressures are compared, as shown in Figure 2.

Figure 2a shows the density distribution of CO_2_ molecules along the *z*-axis at different temperatures and pressures. As the system temperature increases, the density peak of CO_2_ molecules at the gas–liquid interface decreases, and their density values in the aqueous region change very little. The reason for the analysis is that an increase in temperature increases the kinetic energy of CO_2_ molecules, making their thermal motion more intense. The distance between the CO_2_ molecules and water molecules increases, and at this time, the kinetic energy of the CO_2_ molecules themselves is greater than the interaction energy between them and water molecules. Some CO_2_ molecules break free from the binding of dehydrated molecules and diffuse outside the interface (gas phase), resulting in a decrease in the density peak of CO_2_ molecules at the interface. When the gas phase pressure increases from 1 MPa to 3 MPa, the peak density of CO_2_ molecules at the gas–liquid interface increases from 0.04 g/cm^3^ to 0.06 g/cm^3^, indicating that the increase in pressure significantly increases the number of CO_2_ molecules in the CO_2_ adsorption layer; on the one hand, the reason for this is that the higher pressure compresses the volume of the CO_2_ phase, resulting in an increase in the number of CO_2_ molecules per unit volume of the system. On the other hand, it reduces the spatial distance between CO_2_ and water molecules and enhances the interaction between molecules, and more CO_2_ approaches the water phase and adsorbs at the interface. Therefore, an increase in pressure will lead to a significant increase in the peak density of CO_2_ molecules at the interface.

Figure 2b shows the density distribution of water molecules along the *z*-axis at different temperatures and pressures. The peak density of water molecules slightly decreases with increasing temperature, but the change is not significant. It indicates that the temperature fluctuation from 20 K has a small impact on the distribution characteristics of water molecules, the reason for which is that water molecules have strong interactions—not only van der Waals interactions, but also stronger hydrogen-bonding interactions. As a result, the degree of weakened intermolecular interaction energy with increased temperature decreases, so the decrease in water molecule density with increasing temperature is relatively small. As the system pressure increases, the density peak of water molecules slightly decreases. The reason for this is that an increase in pressure reduces the distance between molecules and enhances the interaction force between carbon dioxide and water molecules, and more carbon dioxide molecules enter the water phase, increasing the distance between water molecules and thus reducing the density of water.

**Figure 3:**
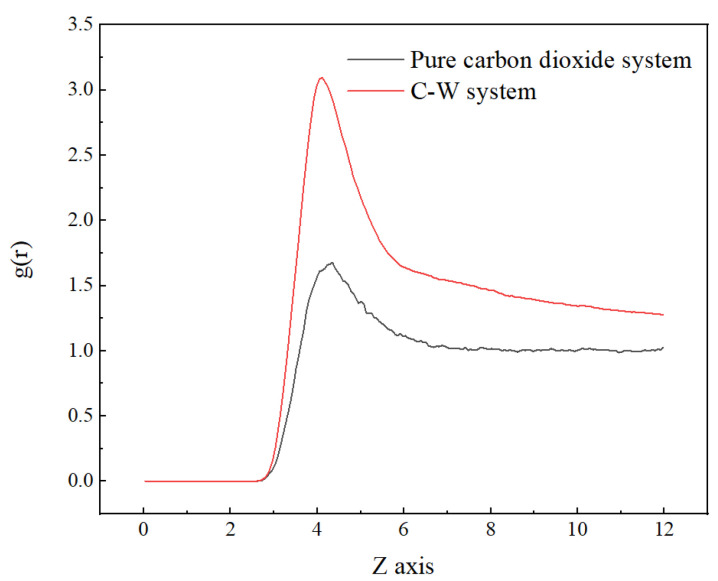
Radial distribution function of CO_2_ (C)–CO_2_ (C) in pure carbon dioxide system and C-W system at 313 K.

Compared with the pure carbon dioxide system, it is found that the RDF peak is located slightly to the left and had a larger peak, as shown in Figure 3, indicating a more compact distribution of carbon dioxide in the two-phase system. This is due to the interaction force between water molecules and carbon dioxide molecules.

**Figure 4:**
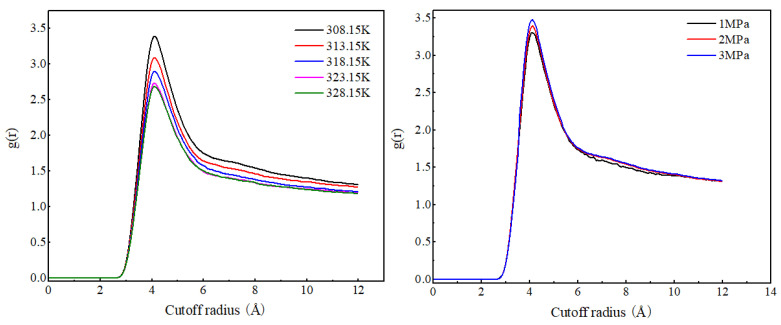
Radial distribution function of CO_2_ (C)–CO_2_ (C) at different temperatures and pressures.

As shown in Figure 4, the radial distribution function curves of CO_2_ molecules at different temperatures have the same shape, with the peak position at 4.11 Å. As the temperature increases, the RDF peak of CO_2_ molecules gradually decreases, indicating that the increase in temperature weakens the degree of the aggregation of CO_2_ molecules. The radial distribution function curve shapes and peak positions of CO_2_ molecules under different pressures are basically the same. As the pressure increases, the RDF peak value of CO_2_ molecules significantly increases, and the increase is much greater than the increase caused by the decrease in temperature. This is because CO_2_ molecules have a low density and strong compressibility. An increase in pressure will significantly reduce the distance between CO_2_ molecules, resulting in a significant increase in the RDF value of CO_2_ molecules.

The diffusion coefficients are calculated using Einstein’s equation. In this paper, the interphase mass transfer characteristics of the C-W system are studied, and the interphase mass transfer occurs in the *z*-axis direction. Therefore, the diffusion coefficients in the *z*-axis direction are calculated. From Table 1, it can be seen that as the temperature increases, the diffusion coefficients of CO_2_ molecules and water molecules in the *z*-axis direction show an overall increasing trend. This is because an increase in temperature intensifies the irregular thermal motion of molecules, thereby enhancing the diffusion ability of both CO_2_ and water molecules. From Table 2, it can be seen that with the pressure increases, the diffusion coefficient of CO_2_ molecules gradually decreases, while the diffusion coefficient of water molecules changes very little within this pressure range. For CO_2_ molecules, as the system pressure increases, the number of CO_2_ molecules per unit volume increases, limiting their movement space and reducing the average distance between molecules, hindering their diffusion. For water molecules, an increase in pressure slightly increases the number of carbon dioxide molecules around them. Under the interaction force between carbon dioxide and water molecules, the degree of polymerization between water molecules is weakened, promoting their diffusion.

### 2.3. Effect of Salt Concentration on Phase Composition Structure and Diffusion Characteristics of C-W Two-Phase System

(1) Phase composition distribution characteristics of C-SW system

**Figure 5:**
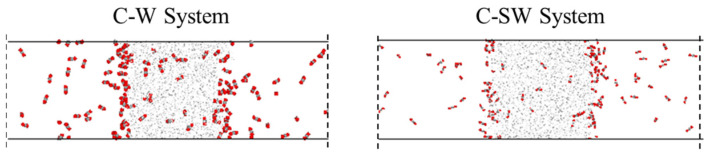
Comparison of mass transfer processes in C-W system and C-SW system.

The presence of salt ions does not affect the direction of diffusion and mass transfer for each component, but it can have an impact on the strength of diffusion and mass transfer. By comparing the final conformations of the C-SW and C-W systems that reach equilibrium in mass transfer, as shown in Figure 5, it can be found that the number of CO_2_ molecules in the aqueous phase of the C-SW system is significantly lower, indicating that ions hinder the diffusion and mass transfer of CO_2_ in the aqueous phase. The reason for the analysis is that ions carry charges, with sodium ions carrying positive charges and chloride ions carrying negative charges, which generate strong electrostatic interactions with water molecules and attract surrounding water molecules to approach them. The sodium ions and chloride ions are surrounded by water molecules, forming a loose hydration layer. At the same time, under the interaction between water molecules, more water molecules arrange around the ions, and the hydration layer becomes tighter and more orderly, forming a stable hydration shell structure. Furthermore, it is difficult for the interaction between the CO_2_ molecules and water molecules to completely damage the hydration shell structure formed by electrostatic and hydrogen bonding, resulting in a decrease in the amount of CO_2_ entering the aqueous phase.

**Figure 6:**
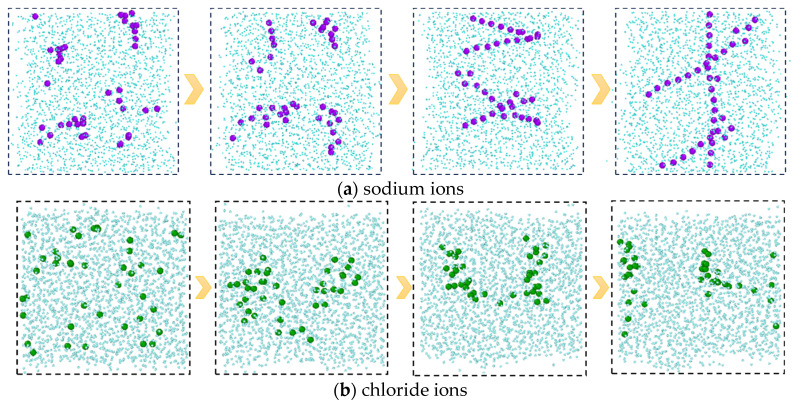
The evolution processes of the ion structures in the C-SW system. (Only the water phase region is shown in the figure, where the carbon dioxide molecules and the hydrogen atoms in the water are deleted).

In addition, from the molecular conformations of each frame, it can be seen that the sodium ions in the C-SW system exhibit a special behavior during this process. In the initial simulation, sodium ions are uniformly distributed in the aqueous phase. Subsequently, the sodium ions transition from an irregular dispersed state to an aggregated state, gradually developing into an ordered state, and finally forming a chain-like structure, as shown in Figure 6, which is a snapshot of the aqueous phase region taken along the xz direction. In order to clearly see the structure of the ions, the image is processed to remove the carbon dioxide molecules and only retain the oxygen atoms in the water molecules. The reason for this phenomenon is that the interaction between the water molecules and the ions generates an equivalent ionic attraction to overcome the strong Coulomb repulsion between ions, resulting in the sodium ions exhibiting a specific hydration structure, namely a highly ordered chain-like structure. However, the chloride ions did not exhibit this special hydration structure clearly. The reason for this is that compared to sodium ions, chloride ions have a larger radius, a lower ion charge density, and weaker interaction with water molecules, which is not sufficient to overcome the Coulombic repulsion between chloride ions.

**Figure 7:**
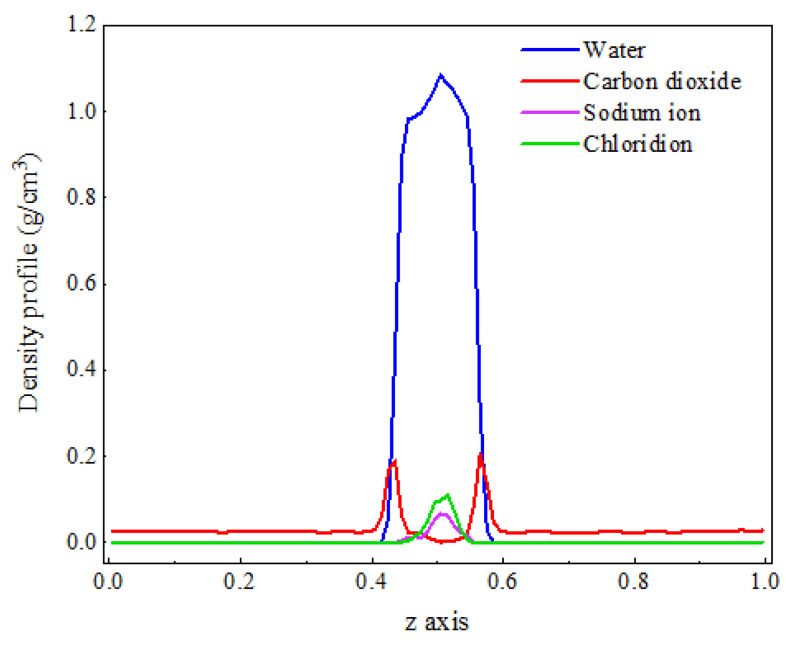
The density distribution of CO_2_ and water molecules at a salt ion concentration of 1.1 M.

Figure 7 shows the density distribution of each component in the C-SW system. It can be observed that the distribution positions of each component in the C-SW and C-W systems are generally consistent, but there is a significant change in their density values. The density of water molecules increases, while the density of CO_2_ molecules in the aqueous phase decreases. This is because the generated hydration structure makes the arrangement of water molecules more compact, increasing the number of water molecules per unit volume, and hindering the entry of CO_2_ molecules into the aqueous phase, which is consistent with the phenomenon exhibited in the mass transfer process.

**Figure 8:**
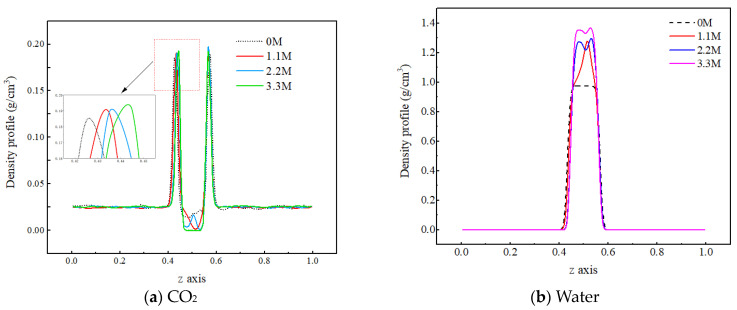
Density distribution of CO_2_ molecules under different salt ion concentrations.

In order to investigate the influence of salt components on the mass transfer process of each component in the C-W system, three concentrations of sodium chloride salt water, namely 1.1 M, 2.2 M, and 3.3 M, are selected for analysis. Firstly, the mechanism by which the concentration of salt components affects the density distribution of each component in the two-phase system is investigated, as shown in Figure 8.

From Figure 8a, it can be observed that as the salt concentration in water increases, the density peak of carbon dioxide molecules at the gas–liquid interface slightly increases, while the density value in the aqueous phase region significantly decreases, indicating that the ions hinder the diffusion of CO_2_ molecules into the water phase. The reason for this is that there is a strong interaction between ions and water molecules. The water molecules form a hydrated shell around the ions, reducing the available free space for CO_2_ molecules in the water phase. In addition, the interaction force between CO_2_ molecules and water molecules is smaller than that between water molecules and ions, making it difficult for the CO_2_ molecules to destroy the structure formed by the water molecules and ions, resulting in the more difficult entry of CO_2_ molecules into the water phase. As shown in Figure 8b, as the salt concentration increases, the density of the aqueous phase increases, which is due to the inherent characteristics of the ions. On the one hand, the mass of the ions is relatively large, and they are evenly distributed in the pores of the water molecules without causing any volume change. As a result, the total mass of particles per unit volume increases, leading to an increase in the density of the water phase. On the other hand, the charged nature of ions causes the water molecules to approach them, forming a tighter structure, which also leads to an increase in the density of the water phase.

**Figure 9:**
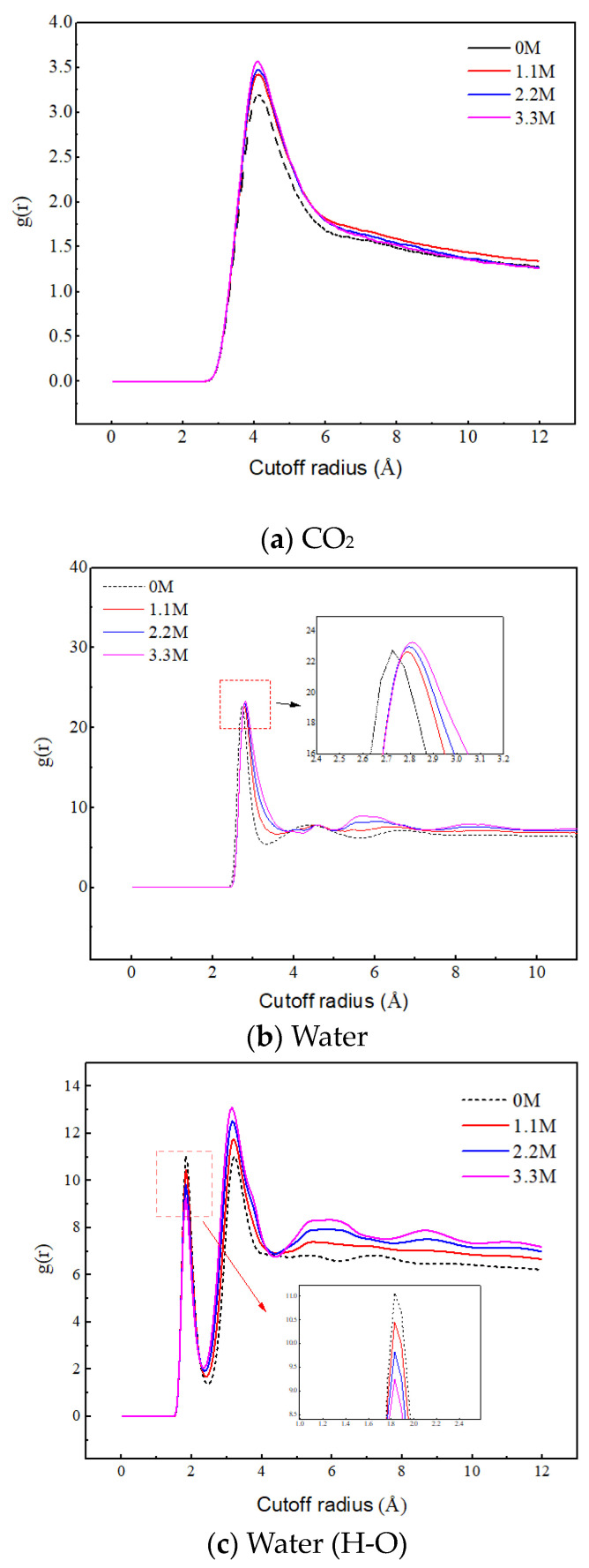
Radial distribution functions of C–C, O–O, and H–O at different ion concentrations.

Figure 9a reveals the effect of salt ion concentration on the aggregation behavior of CO_2_. As the concentration of salt ions increases, the peak value between CO_2_ molecules significantly increases, indicating that the increase in ion concentration promotes the aggregation of CO_2_. This is because the increase in ion concentration leads to the enhancement of the ion hydration layer structure, and the water molecules are firmly bound by the ions, thereby weakening their attraction to CO_2_. Therefore, the CO_2_ molecules are mainly distributed in the gas phase region, and their quantity significantly increases, leading to a significant increase in their aggregation degree.

Figure 9b,c reveal the effect of salt ion concentration on the aggregation behavior of water molecules. The position corresponding to the first peak in Figure 9b represents the distance between the oxygen atom of the central water molecule and the oxygen atom of the nearest neighbor water molecule. In Figure 9c, the position corresponding to the first peak represents the distance of hydrogen bonding, and the position corresponding to the second peak represents the distance between the hydrogen atom of the central water molecule and the oxygen atom of the nearest neighbor water molecule without hydrogen bonding. With the increase in salt ion concentration, the RDF peak of O–O in water molecules increases, and the second peak of RDF of H–O increases, both of which indicate that the increase in salt ion concentration promotes the aggregation of water molecules. The position of the RDF peak of O–O shifts with the increase in salt concentration and the first peak of the RDF of H–O decreases with the increase in salt concentration, which indicates that the ions disrupt the original hydrogen bonding network between the water molecules. The sodium ions intervene between the water molecules, and under electrostatic action, the water molecules gather more tightly around the ions and extend outward to form a new hydrogen bonding network. In addition, the increase in the oscillation amplitude of RDF indicates that the ions enhance the long-range ordering of the water molecules, leading to a more ordered distribution of water molecules. These results indicate that salt ions play a crucial role in regulating the aggregation behavior of water molecules.

**Figure 10:**
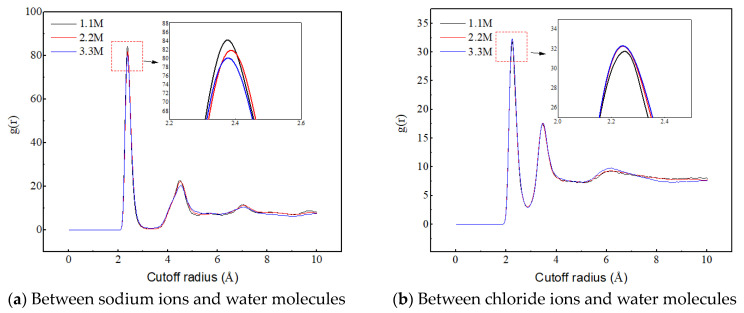
Radial distribution functions between sodium ions, chloride ions, and water molecules at different ion concentrations.

**Figure 11:**
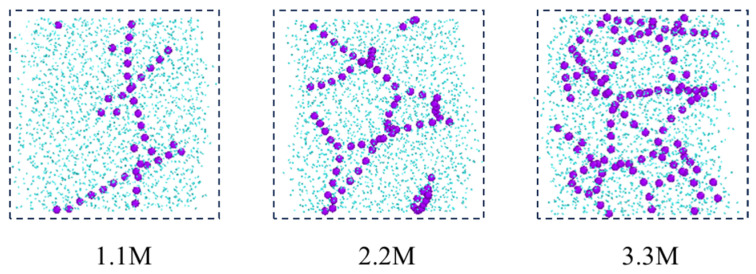
Distribution of sodium ions after reaching equilibrium at different salt concentrations (purple ball: sodium ion; blue ball: water).

Figure 10a reveals the influence of salt ion concentration on the structure and quantity of sodium ion hydrated shells, indicating that salt ion concentration has no effect on the structure of hydrated shells. The first hydrated shell is formed at 2.375 Å and the second hydrated shell is formed at 4.525 Å. However, as the salt ion concentration increases, the RDF peak decreases, indicating a decrease in the number of hydrated shells around the sodium ions. The reason for this is that the increase in salt ion concentration causes the sodium ion-specific hydration structure to transform from a chain-like structure to a network-like structure, as shown in Figure 11. The complex network structure hinders the hydrated shells of the water molecules from developing into multiple layers, resulting in a decrease in the number of hydrated shells around the sodium ions and a decrease in the peak values of the radial distribution functions between the sodium ions and the water molecules.

Figure 10b reveals the influence of salt ion concentration on the structure and quantity of chloride ion hydrated shells, indicating that the concentration of salt ions has no effect on the structure of chloride ion hydrated shells. The first hydrated shell is formed at 2.225 Å, and the second hydrated shell is formed at 3.475 Å. However, as the concentration of salt ions increases, the RDF peak increases, which is completely different from the RDF peak between sodium ions and water molecules that changes with salt ion concentration. This is because chloride ions do not form a special hydration structure, and the increase in ion concentration enhances the probability of interaction between chloride ions and water molecules, thereby producing more hydrated shells.

The presence of salt ions also affects the diffusion ability of CO_2_ and water molecules. Table 3 shows the diffusion coefficients of CO_2_ and water molecules in the *z*-axis direction at different salt ion concentrations. The results show that the diffusion coefficients of carbon dioxide and water decrease with the increase in salt ion concentration. The reason for the decrease in the diffusion coefficient of carbon dioxide is that the hydration of the salt ions and water molecules inhibits the diffusion of carbon dioxide molecules in the aqueous phase, exhibiting salting out characteristics. The decrease in the diffusion coefficient of water is due to the strong interaction between ions and water molecules, and sodium ions form a special hydration structure. The water molecules cannot undergo disordered thermal motion, but instead move in synergy with the ions, resulting in a significant weakening of the diffusion ability of the water molecules.

## 3. Model Establishment and Simulation Details

**Figure 12:**
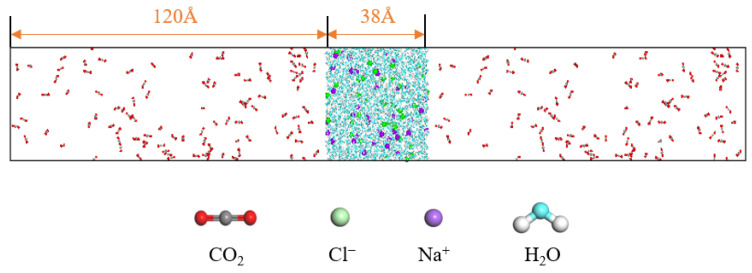
C-SW model.

In order to investigate the diffusion and mass transfer characteristics between carbon dioxide and water molecules, a two-phase model of carbon dioxide and water (C-W model) is constructed. The water molecules are in the middle of the box, while the CO_2_ molecules are distributed on both sides of the box. The ratio of CO_2_ to water molecules satisfies the condition that the number of CO_2_ molecules is greater than the amount dissolved in water, resulting in a clear gas–liquid interface in the system. The system pressure is changed by changing the number of gas molecules in the system. The pressure of the gas can be obtained by the Peng–Robinson equation. There are a large number of mineral ion components in the extracted water, and salt components have a significant impact on the properties of the water. It is necessary to consider the influence of salt on the diffusion and mass transfer characteristics of the CO_2_–water system. The concentration of sodium and chloride ions in the mineral ions of water is relatively high, so in this paper, sodium and chloride ions as salt components are selected. On the basis of the C-W model, a CO_2_–saline water model (C-SW model) is constructed, as shown in Figure 12.

The CO_2_ molecule is modeled using the EPM2-FLEX force field, which incorporates full flexibility [23,24,25]. The water molecule is described by the TIP4P/2005 model, a fully atomistic polarizable charge-distribution water model proposed by J. L. F. Abascal and C. Vega in 2005. The TIP4P/2005 model, which is an extension of the original three-point model, introduces a virtual charged atom to improve the charge distribution, and requires the maintenance of bond lengths and angles during calculations [26,27,28]. These two models have been used in the previous research results, and their force field parameters refer to the reference [29]. The force field parameters of sodium ions and chloride ions refer to Reference [30]. Both the C-W and C-SW systems are subjected to 10 ns molecular dynamics simulations in the NVT ensemble, with the last 2 ns of simulation data used for statistical analysis. The time step is set to 1 fs, and the atomic positions and structural information are output every 10,000 steps. The Nose–Hoover thermostat method is used to control the temperature. Periodic boundary conditions are applied, with a cutoff radius of 12 Å. The simulation conditions are set according to the actual operating conditions of oil and gas transportation, with temperatures ranging from 308.15 K to 328.15 K and pressures from 1 MPa to 3 MPa. This simulation process is realized through Material Studio 2017 and Lammps 2020.

## 4. Conclusions

In this paper, the molecular dynamics method is used to study the interphase mass transfer and phase composition distribution characteristics of C-W/SW systems under gathering and transportation conditions, as well as the influence mechanism of different temperatures, pressures, and salt ion concentrations on the interphase mass transfer characteristics and phase composition distribution characteristics of the system. The following conclusions are drawn:

(1) The interphase mass transfer process of the C-W system under gathering conditions is divided into three processes: ① CO_2_ molecules gradually move towards the water phase; ② CO_2_ molecules adsorb to the gas–liquid interface and form a loose adsorption layer with a certain thickness and structure; ③ a small amount of CO_2_ molecules enter the water phase, occupying part of the free space in the water phase.
(2) The influence mechanism of temperature and pressure on the parameters describing the structural and mass transfer characteristics of the system was obtained. As the system temperature increases and pressure decreases, the density peak of CO_2_ molecules at the gas–liquid interface significantly decreases, the RDF peak gradually decreases, and the self-diffusion coefficient gradually increases. The analysis is that both the increase in temperature and the decrease in pressure weaken the interaction energy between CO_2_ molecules and water molecules, and some CO_2_ molecules will break free from the binding of water molecules, resulting in a decrease in the density peak of CO_2_ molecules at the interface, a weakening of the degree of aggregation, and an increase in diffusion ability.
(3) The addition of salt ions to water exhibits a specific hydration structure, and it is difficult for the interaction between CO_2_ molecules and water molecules to completely damage the hydration shell structure formed by electrostatic and hydrogen bonding effects, resulting in a decrease in the amount of CO_2_ entering the aqueous phase. Moreover, as the salt concentration in water increases, the density peak of carbon dioxide molecules at the gas–liquid interface slightly increases, while the density value in the water phase region significantly decreases. At the same time, the RDF peak between carbon dioxide molecules significantly increases, resulting in an increase in aggregation.

## Figures and Tables

**Table 1:** The diffusion coefficients of each component in the *z*-axis direction at different temperatures.

Temperature (K)	308.15	313.15	318.15	323.15	328.15
The diffusion coefficient of CO_2_ (10^−9^ m^2^/s)	4.7	5.6	6.1	5.8	6.2
The diffusion coefficient of water (10^−11^ m^2^/s)	1.3	3.0	8.5	9.0	9.1

**Table 2:** The diffusion coefficients of each component in the *z*-axis direction under different pressures.

Pressure (MPa)	1	2	3
The diffusion coefficient of CO_2_ (10^−9^ m^2^/s)	9.4	4.7	4.4
The diffusion coefficient of water (10^−11^ m^2^/s)	1.1	1.3	2.4

**Table 3:** The diffusion coefficients of each component in the *z*-axis direction under different salt ion concentrations.

Ion concentration (M)	1.1	2.2	3.3
The diffusion coefficient of CO_2_ (10^−10^ m^2^/s)	4.8	1.3	0.6
The diffusion coefficient of water (10^−12^ m^2^/s)	7.9	3.8	3.5

## Data Availability

Data are contained within the article.
